# *Dolosigranulum pigrum*: A promising nasal probiotic candidate

**DOI:** 10.1371/journal.ppat.1011955

**Published:** 2024-02-01

**Authors:** Reed M. Stubbendieck, Jillian H. Hurst, Matthew S. Kelly

**Affiliations:** 1 Department of Microbiology and Molecular Genetics, Oklahoma State University, Stillwater, Oklahoma, United States of America; 2 Department of Pediatrics, Division of Infectious Diseases, Duke University School of Medicine, Durham, North Carolina, United States of America; 3 Children’s Health and Discovery Institute, Department of Pediatrics, Duke University School of Medicine, Durham, North Carolina, United States of America; 4 Department of Molecular Genetics and Microbiology, Duke University School of Medicine, Durham, North Carolina, United States of America; University of Maryland, Baltimore, UNITED STATES

Acute respiratory infections (ARIs) are a major cause of morbidity and mortality across the life span. Globally, an estimated 17 billion ARIs occur each year, accounting for 2.4 million deaths (>740,000 deaths among children) [1]. Although the majority of these ARIs are caused by respiratory viruses, most severe or fatal cases are caused by bacterial respiratory pathobionts. Increasingly, commensal microbes in the upper respiratory tract (URT) are recognized to influence the risk and severity of respiratory viral infections and resistance to colonization and infection by bacterial pathobionts. Consequently, there is growing interest in leveraging these microbe–microbe or microbe–host interactions to develop novel strategies for ARI prevention or treatment [2]. Although the modern history of probiotics dates back more than a century, intranasal administration of live bacterial strains would represent a shift in our approach to preventing and treating ARIs. Necessary characteristics of such nasal probiotics would include the ability to adhere to the epithelium and successfully colonize the human URT, a lack of cytotoxicity to respiratory epithelial cells, some degree of resistance to horizontal gene transfer and mobile genetic elements, a low propensity to invade host tissues, and susceptibility to commonly available antibiotics. Below, we describe an understudied bacterial species, *Dolosigranulum pigrum*, which is increasingly viewed as a keystone species within the human URT and a promising nasal probiotic candidate for ARI prevention or treatment.

## What is *D. pigrum*?

Deriving its name from the Latin words *dolosus* (“crafty, deceitful”), *granulum* (“a small grain”), and *pigrum* (“lazy”), *D. pigrum* (phylum: Firmicutes, class: Bacilli, order: Lactobacillales, family: Carnobacteriaceae) is a gram-positive, lactic acid bacterium that was first described by Aguirre and colleagues in 1993 [3]. It is non-spore-forming, facultatively anaerobic, catalase-negative, and generally susceptible to beta-lactams, clindamycin, and other commonly used antibiotics [4,5]. *D. pigrum* is the only recognized species within the genus *Dolosigranulum*. Its nearest phylogenetic relative is the putative otopathogen *Alloiococcus otitis*; the genetic similarity of these 2 species frequently resulted in taxonomic misclassification in analyses using 16S ribosomal RNA gene sequences from older reference databases [6]. *D. pigrum* has a relatively small genome size (<2 Mb for most strains) and has predicted auxotrophies for several amino acids, polyamines, and enzymatic cofactors, suggesting that it relies on its host or other microbes to provide these nutrients [7]. A recent analysis of *D. pigrum* strains collected over a 20-year period reported that the genomes of these strains were remarkably stable over time and possessed highly conserved chromosomal synteny [8]. Further, these genomes were found to contain multiple genes encoding clustered regularly interspaced short palindromic repeats (CRISPRs) and restriction–modification systems that may serve to limit horizontal gene transfer and exclude mobile genetic elements [8].

### What are the natural habitats of *D. pigrum*?

*D. pigrum* is highly adapted to the human nasal passages. In an analysis of 8,184 samples from 6 human body sites, *Dolosigranulum* sequencing reads were identified in 41% of nasal samples, 15% of skin samples, and <1% of fecal and oral cavity samples [9]. Moreover, in samples in which *Dolosigranulum* was detected, the organism was far more abundant in nasal samples (18% mean relative abundance) than in samples from other body sites (<2% mean relative abundance) [9]. Analyses of data from the Earth Microbiome Project revealed that *Dolosigranulum* reads were rarely identified in environmental sources (e.g., water, soil) but were identified in samples from a variety of animal species, including rodents, fish, birds, dogs, and primates [9]. Notably, *Dolosigranulum* was generally of low abundance in the microbiomes of these animals, although relative abundances approaching 50% were observed in some dogs [9]. Within humans, the prevalence and abundance of *D. pigrum* in the URT varies markedly across the life span. The mean relative abundance of *Dolosigranulum* in the nasal passages increases progressively during infancy (from approximately 1% in the days after birth to 10% to 20% by 12 months of age), remains largely stable during childhood, and declines during adolescence, coinciding with pubertal development [10–12]. Although comparatively few studies of the URT microbiota have been conducted among healthy adults and the elderly, nasal abundances of *Dolosigranulum* in these populations have generally been reported to be lower than those observed in children [12,13].

### Is *D. pigrum* a human pathogen or pathobiont?

The original description of *D. pigrum* included 2 strains, one of which was isolated from postmortem human spinal cord tissue and the second of which was cultivated from eye and contact lens cultures from a woman with blurred vision and eye pain [3]. This description, along with several subsequent reports that similarly described instances in which *D. pigrum* was cultivated from patients with infections, suggested that this bacterium may have a role as a pathobiont [5,14]. However, more recent studies suggest that *D. pigrum* has limited pathogenicity in humans. The bacterium is now recognized to be almost ubiquitously present in the human URT, particularly during infancy and early childhood, and yet has only rarely been implicated in human infections, typically among elderly adults with compromised immunity [3,14–18]. Moreover, on many of the occasions when *D. pigrum* has been cultured from patients with infections, it has often grown in mixed cultures with established pathobionts, making its role in these infections uncertain [3,9]. Notably, none of the 34 *D. pigrum* genomes currently available in GenBank harbor genes encoding proteins that are closely related to known virulence factors, and *D. pigrum* has not been found to be cytotoxic to human respiratory epithelial cells in ex vivo experiments [9,19].

### Is *D. pigrum* beneficial to human respiratory health?

Recent studies suggest that the presence or abundance of *D. pigrum* in the URT microbiota is associated with various states of respiratory health (**Table 1**). Compared to healthy controls, lower URT abundances of *D. pigrum* have been observed among infants and children with ARIs, children with acute otitis media, and adults with chronic rhinosinusitis [9,20–23]. Moreover, *D. pigrum* has been associated with the absence of URT colonization by bacterial respiratory pathobionts. Most notably, lower relative abundances of *D. pigrum* are found in the nasal cavities of *Staphylococcus aureus* carriers [4,24–27], and a prospective study found that higher nasal abundances of *D. pigrum* were associated with a lower risk of *Staphylococcus aureus* acquisition during infancy [28]. *D. pigrum* may also influence the outcome of efforts to decolonize *Staphylococcus aureus* carriers. Use of nasal mupirocin can eliminate both *Staphylococcus aureus* and *D. pigrum* from the nasal microbiota, while recolonization with *Staphylococcus aureus* after stopping mupirocin is associated with delayed recolonization by *D. pigrum* [4]. Several cross-sectional studies also reported lower relative abundances of *D. pigrum* among children colonized by *Streptococcus pneumoniae*, a bacterial pathobiont that is among the most common bacterial causes of pneumonia, acute otitis media, and acute bacterial rhinosinusitis [20,29]. Finally, compared to other nasal microbiome profiles, a profile codominated by *Corynebacterium* and *Dolosigranulum* was associated with a lower risk of respiratory viral infections during infancy [30].

**Table 1:** Associative microbiome studies supporting the role of *Dolosigranulum pigrum* as a mutualist in the human upper respiratory tract.

Author, Year	Population	Sample Type	Association
Laufer, 2011 [20]	Children (<7 y)	Nasal swab	↓ abundance in acute otitis media ↓ abundance with *Streptococcus pneumoniae* carriage
Liu, 2015 [24]	Adults (>18 y)	Nasal swab	↓ abundance with *Staphylococcus aureus* carriage
Escapa, 2018 [27]	Adults (18–40 y)	Nasal swab	
Khamash, 2019 [26]	Neonates	Nasal swab	
Wagner Mackenzie, 2021 [25]	Adults (>18 y)	Nasal swab	
Accorsi, 2020 [28]	Infants (<1 y)	Nasal swab	↓ abundance with increased risk of *Staphylococcus aureus* acquisition
Teo, 2015 [21]	Infants (<1 y)	NP aspirate	↓ abundance with acute respiratory infection symptoms
Kelly, 2017 [22]	Children (<2 y)	NP swab	
Hasegawa, 2017 [31]	Children (<5 y)	Nasal swab	
de Koff, 2021 [32]	Children (<2 y)	NP swab	
de Steenhuijsen Piters, 2022 [30]	Children (<2 y)	NP swab	
Lappan, 2018 [23]	Children (<5 y)	NP swab	↓ abundance in acute otitis media
Gan, 2019 [33]	Adults (>18 y)	Nasal swab	↓ abundance in chronic rhinosinusitis
De Boeck, 2021 [9]	Adults (18–65 y)	NP swab	
Tang, 2021 [34]	Children (<2 y)	Nasal lavage	↓ abundance with increased risk of wheezing illness
Patel, 2023 [29]	Children (<5 y)	NP swab	↓ abundance with *S. pneumoniae* colonization

NP, nasopharyngeal

Relatively little is known regarding the mechanisms by which *D. pigrum* promotes human respiratory health and interacts with other microbes and host cells within the human URT. Consistent with findings from epidemiological studies, some *D. pigrum* strains demonstrate in vitro growth inhibition of *Staphylococcus aureus*, although efforts to identify the underlying inhibitory mechanisms have thus far been unsuccessful [7,9,25]. *D. pigrum* may also act in concert with other URT commensals. While growth of *S. pneumoniae* is not inhibited on solid medium conditioned with *D. pigrum* or *Corynebacterium pseudodiphtheriticum* alone, inhibition is observed on medium conditioned by cocultivation of these species [7]. Similar to other lactic acid bacteria, *D. pigrum* produces L-lactic acid in millimolar concentrations in vitro, spurring interest in determining the extent to which lactic acid production by *D. pigrum* contributes to its interactions with other URT microbes [7,9]. Notably, the concentrations of lactic acid produced by *D. pigrum* are insufficient to inhibit in vitro growth of *Staphylococcus aureus* or *S. pneumoniae*, although similar concentrations have been reported to inhibit growth of the respiratory pathobiont *Moraxella catarrhalis* [35]. Ex vivo and in vivo models have provided additional insights into *D. pigrum* and its interactions with the host that may mediate protection from infections by respiratory pathobionts. Exposing human airway epithelial cells to *D. pigrum* reduced up-regulation of genes encoding the proinflammatory cytokines interleukin (IL)-1β and IL-8 in response to *Staphylococcus aureus* exposure, suggesting that *D. pigrum* reduces the tissue-damaging inflammation induced by this pathobiont [9]. Similarly, larvae of the greater wax moth died rapidly following injection of *Staphylococcus aureus*, whereas improved survival was observed when *D. pigrum* was coinjected with *Staphylococcus aureus* [9]. In mice, intranasal administration of *D. pigrum* prior to *S. pneumoniae* challenge reduced lung pneumococcal cell counts, promoted earlier recruitment of innate immune cells, and resulted in lower alveolar levels of proinflammatory cytokines and markers of tissue injury [36]. Finally, nasally administered *D. pigrum* altered respiratory and systemic cytokine profiles and lowered alveolar viral loads in mice experimentally infected with RSV [37].

### Future directions

In conclusion, *D. pigrum* is an understudied lactic acid bacterium that is increasingly viewed as a mutualist in the human URT. Although early reports suggested that this species may act as a pathobiont, more recent studies demonstrate that it is a highly prevalent member of the URT microbiota that has only rarely been cultivated from individuals with infections, often in mixed cultures with established human pathobionts. *D. pigrum* appears to exert its beneficial effects in the human respiratory tract through both direct interactions with bacterial respiratory pathobionts and modulation of host immune and inflammatory responses. Given its multiple predicted auxotrophies and potential dependence on interactions with other commensal bacteria to produce some antimicrobial factors, *D. pigrum* may not be effective as a single-strain probiotic but rather may require administration in a probiotic consortium to confer health benefits. Regardless, though further research is needed, *D. pigrum* possesses several key characteristics that make it a highly promising nasal probiotic candidate for the prevention or treatment of ARIs and the promotion of human respiratory health.
